# Natural Feed Additives Modulate Immunity and Mitigate Infection with *Sphaerospora molnari* (Myxozoa: Cnidaria) in Common Carp: A Pilot Study

**DOI:** 10.3390/pathogens9121013

**Published:** 2020-12-02

**Authors:** Vyara O. Ganeva, Tomáš Korytář, Hana Pecková, Charles McGurk, Julia Mullins, Carlos Yanes-Roca, David Gela, Pavel Lepič, Tomáš Policar, Astrid S. Holzer

**Affiliations:** 1 Biology Center of the Czech Academy of Sciences, Institute of Parasitology, 37005 České Budějovice, Czech Republic; vyara.ganeva@gmail.com (V.O.G.); tkorytar@paru.cas.cz (T.K.); pecka@paru.cas.cz (H.P.); 2 South Bohemian Research Center of Aquaculture and Biodiversity of Hydrocenoses, Faculty of Fisheries and Protection of Waters, University of South Bohemia, 37005 České Budějovice, Czech Republic; cyanesroca@frov.jcu.cz (C.Y.-R.); gela@frov.jcu.cz (D.G.); lepic@frov.jcu.cz (P.L.); policar@frov.jcu.cz (T.P.); 3 Skretting Aquaculture Research Centre, 4016 Stavanger, Norway; Charles.McGurk@skretting.com (C.M.); Julia.Mullins@skretting.com (J.M.)

**Keywords:** in-feed, curcumin, yeast, parasite, fish, innate immunity, adaptive immunity

## Abstract

Myxozoans are a diverse group of cnidarian parasites, including important pathogens in different aquaculture species, without effective legalized treatments for fish destined for human consumption. We tested the effect of natural feed additives on immune parameters of common carp and in the course of a controlled laboratory infection with the myxozoan *Sphaerospora molnari*. Carp were fed a base diet enriched with 0.5% curcumin or 0.12% of a multi-strain yeast fraction, before intraperitoneal injection with blood stages of *S. molnari*. We demonstrate the impact of these treatments on respiratory burst, phagocytosis, nitric oxide production, adaptive IgM+ B cell responses, *S. molnari*-specific antibody titers, and on parasite numbers. Both experimental diets enriched B cell populations prior to infection and postponed initial parasite proliferation in the blood. Curcumin-fed fish showed a decrease in reactive oxygen species, nitric oxide production and B cell density at late-stage infection, likely due to its anti-inflammatory properties, favoring parasite propagation. In contrast, multi-strain yeast fraction (MsYF)-fed fish harbored the highest *S. molnari*-specific antibody titer, in combination with the overall lowest parasite numbers. The results demonstrate that yeast products can be highly beneficial for the outcome of myxozoan infections and could be used as effective feed additives in aquaculture.

## 1. Introduction

Myxozoans are cnidarian endoparasites with reduced morphology and commonly a two-host life cycle, usually including fish as vertebrate hosts. Some of the most severe fish diseases caused by myxozoans include proliferative kidney disease and whirling disease in salmonids [1,2], proliferative gill disease in channel catfish [3] and emaciation disease caused by *Enteromyxum* spp [4,5]. Myxozoans can have a significant impact on aquaculture and wild fish, resulting in substantial economic losses [6]. Critically, with the increase of water temperatures, epidemiological models predict spreading of myxozoans into new geographic regions and disease outbreaks [7]. A northwards migration trend of *Tetracapsuloides bryosalmonae* was confirmed by several records of disease outbreaks from European territories [8,9,10,11] and recent reports exist of massive fish mortalities from natural habitats such as the Yellowstone river [12,13]. In Central Europe, rising water temperatures have been associated with increased numbers of proliferative stages of *Sphaerospora molnari* in the blood of common carp *Cyprinus carpio* [14].

Increasing water temperatures and the growing impact of the aquaculture industry call for action in disease prevention and treatment. Unfortunately, despite progress in the understanding of disease pathology and immune protection in various species, there is currently no treatment for these parasites, and substantial progress towards future protective vaccines has been made only in a handful of fish species. It is therefore critical to explore alternative antiparasitic strategies. Chemotherapeutants such as ivermectin and its derivatives are extensively used against a variety of parasites in both farm animals and humans [15]. However, in addition to the emerging resistance in some treated parasites, these compounds accumulate in the environment, where they can potentially be toxic to other species [15,16]. Therefore, they hardly represent a good alternative for the aquaculture industry.

Nutraceuticals seem a promising alternative since they are based on natural compounds which often present no toxicity and no risk of persistence of chemical residues in fish tissues or the environment, and they are mostly delivered as in-feed applications without major intervention (stress induced by injections, bath treatments, etc.) Additionally, a wide range of natural compounds exist, some targeting pathogens, others positively modulating host immune responses. Reports on the use of feed additives against myxozoans are scarce but provide promising outlooks. Experimental evidence showed that feeding with yeast-derived β-glucans decreases the number of *Ceratomyxa shasta* parasite stages in rainbow trout (*Oncorhynchus mykiss*), despite an unchanged overall survival rate [17]. Furthermore, a recent study by Palenzuela et al. [18] describes the positive impact of the functional feed additive SANACORE^®^ GM on the prevalence and intensity of *Enteromyxum leei* infection in gilthead sea bream (*Sparus aurata*).

Based on these reports, we used our in-house myxozoan in vivo transmission and infection model, *S. molnari* in common carp, to provide insights into the protective capacity of in-feed additives whose effect had previously not been studied in myxozoan parasites. More specifically, we investigated how curcumin, a parasiticidal agent with known anti-inflammatory activity and a multi-strain yeast fraction (MsYF), an immunomodulator, influence the innate and adaptive immune responses of common carp and whether the effect translates into a reduction in the number of proliferative blood stages of *S. molnari* and specific antibodies raised against it.

## 2. Results

### 2.1. Diets Induce Similar Weight Gain in All Investigated Groups

**Figure 1:**
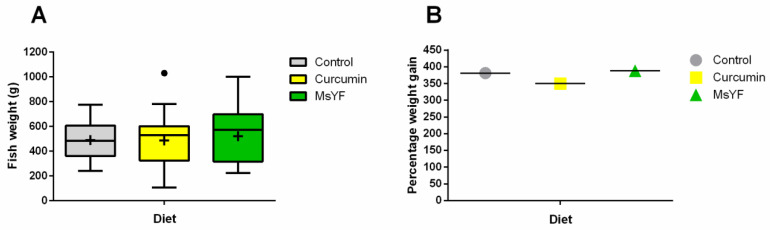
Effect of curcumin- and MsYF-based diets on fish weights. (**A**) Common carp (*Cyprinus carpio*) body weight at the end of the 106-day trial (two months of feeding and 1.5 months of infection with *S. molnari*). Mean values are indicated by a “+” sign, medians by a line. (**B**) Average percentage of weight gain of non-infected fish during the 106-day trial.

Throughout the whole experiment, fish feeding response and feed intake were high and all food was consumed across test groups, at each meal. Fish weights of non-infected individuals after 106 days of feeding (42 dpi) did not show significant differences between experimental groups; however, the MsYF-supplemented group contained several individuals of higher body weight when compared to the control (Figure 1A). The percentage weight gain during the whole study did not differ significantly between groups (control: 381%, curcumin: 350%, MsYF: 388%; Figure 1B).

### 2.2. Functional Diets Induce Changes in the Immune Parameters of Uninfected Carp

**Figure 2:**
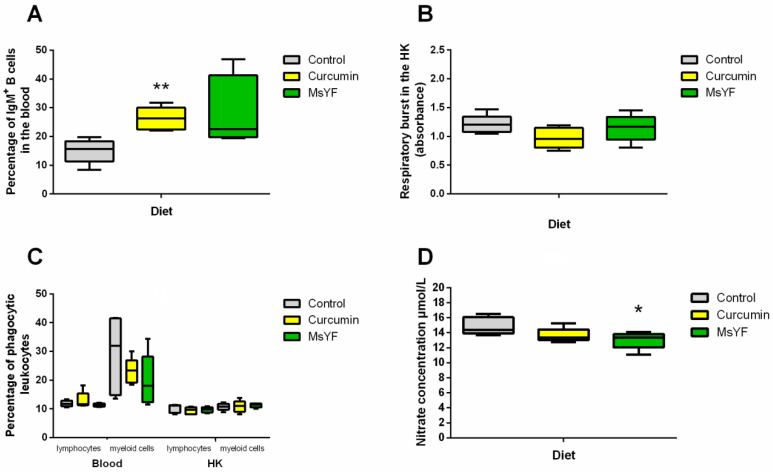
Feed-based changes in immune parameters of common carp (prior to infection with *S. molnari*). (**A**) Percentage of IgM+ cells in the blood. (**B**) Respiratory burst activity of head kidney (HK) leukocytes. (**C**) Percentage of phagocytic leukocytes in the blood and HK. (**D**) Nitric oxide secretion by HK leukocytes. Significant differences between control and experimental diets are indicated by * (*p* < 0.05) and ** (*p* < 0.01) (Kruskal-Wallis test), *n* = 5.

Firstly, we aimed at elucidating the impact of the functional feeds on the innate and adaptive immunity of common carp, prior to the infection with *S. molnari*. We observed a marked increase in the proportion of IgM+ B cells in the blood in fish fed both experimental feeds, with 10.7% and 7.0% higher median values for curcumin and MsYF, respectively, in comparison to the control. However, the values reached statistical significance only in the group of fish fed curcumin (*p* < 0.01), showing lower dispersion compared to MsYF (Figure 2A). We observed a marginal reduction in respiratory burst in curcumin-fed fish in comparison to the control (Figure 2B) and a minor effect on the phagocytic properties of fish leukocytes in blood or head kidney (HK) (Figure 2C). Similarly, both curcumin and MsYF reduced NO secretion in the head kidney leukocytes of naïve fish in comparison to the control slightly; however, the results reached statistical significance only in the MsYF-supplemented group (*p* < 0.05) where median nitrate concentration was 13.4 µmol/L in comparison to 14.4 µmol/L in the control group (Figure 2D). Taken together, these data suggest only a subtle impact of the enriched feeds on innate immune parameters in naïve fish, while IgM+ B cell numbers increased considerably.

### 2.3. Myxozoan Infection Causes Differential Responses in Carp Fed Curcumin vs. Yeast-Enriched Diets

**Figure 3:**
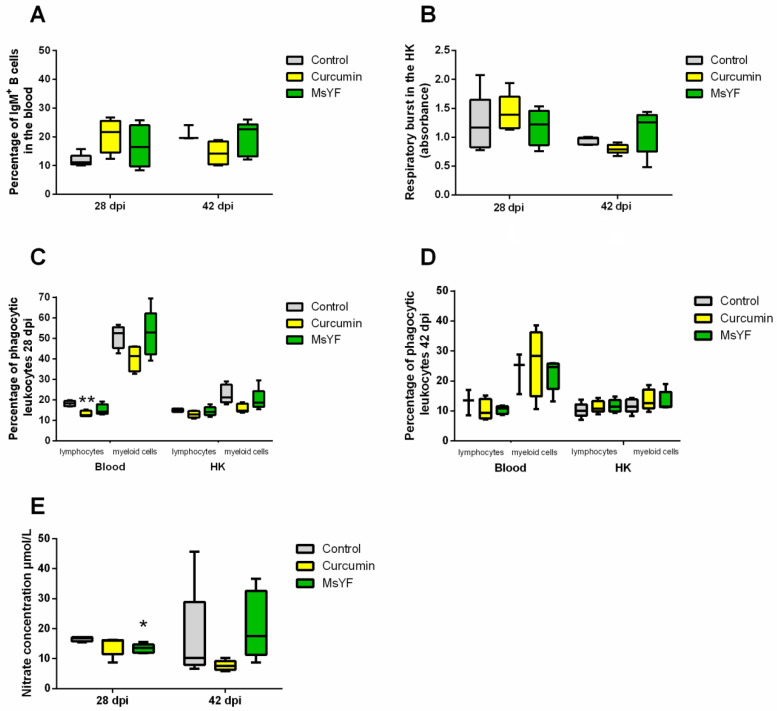
Immune parameters of common carp, 28 and 42 dpi with *S. molnari*. (**A**) Percentage of IgM+ cells in the blood. (**B**) Respiratory burst activity of HK leukocytes. (**C**) Percentage of phagocytic leukocytes in the blood and HK, 28 dpi. (**D**) Percentage of phagocytic leukocytes in the blood and HK, 42 dpi. (**E**) Nitric oxide secretion by HK leukocytes. Significant differences between control and experimental diets are indicated by * (*p* < 0.05) and ** (*p* < 0.01) (Kruskal-Wallis test), *n* = 5, apart from control (**A**) 42 dpi and control blood (**D**) where *n* = 3.

Next, we sought to investigate the effect of the *S. molnari* infection on carp immune response and whether the diets can modulate it. The same immune parameters were measured 28 and 42 days after intraperitoneal injection of the parasite (Figure 3).

The median percentage of IgM+ B cells in the blood was increased 28 dpi with *S. molnari* (10.6% and 5.4% in curcumin and MsYF, respectively), thereby matching the pre-infection status. At 42 dpi, the median value of the proportion of IgM+ B cells was 5.3% lower in the curcumin-supplemented group, but 3.1% higher in the MsYF group, compared with the control (Figure 3A), showing that only the MsYF diet supports a long-term increase in B cell numbers.

In contrast to naive fish (pre-challenge), respiratory burst activity of HK leukocytes in the curcumin-supplemented group was enhanced at 28 dpi, followed by a mild reduction at 42 dpi (Figure 3B). Individuals in the MsYF-supplemented group showed an increased respiratory burst activity relative to the control, 42 dpi. This likely represents an activation of phagocytotic cells based on specific antibody labelling of parasite antigens, in contrast to an innate phagocytotic activity.

Phagocytosis was reduced in the blood of the curcumin-supplemented group, 28 dpi, where the median percentages were 5.7% and 11.1% lower than the control for phagocytic lymphocytes (*p* < 0.01) and myeloid cells, respectively, and with a similar trend in the HK, at that time point (Figure 3C). There were no significant differences in the phagocytic activity, neither in blood nor in HK, at 42 dpi (Figure 3D). As observed in naïve fish, NO secretion by HK macrophages was significantly reduced in the group fed MsYF, 28 dpi (*p* < 0.05), where the median value was 3.3% lower than control (Figure 3E).

### 2.4. Functional Diets Decrease S. molnari Numbers and Enhance Specific Immunity

**Figure 4:**
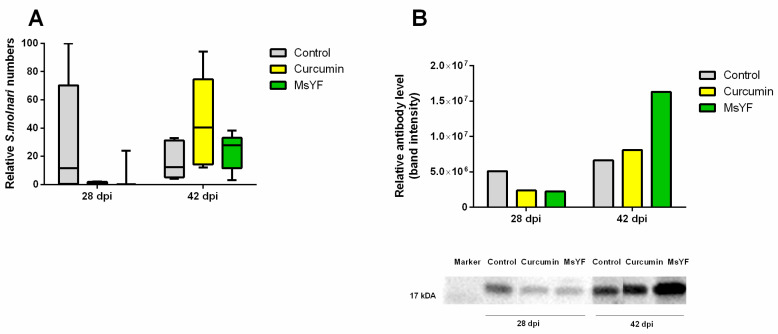
*S. molnari* quantification and serum antibody response. (**A**) *S. molnari* quantities in the blood; values are relative to the sample with the highest value (100, a sample from control diet, 42 dpi). (**B**) Specific antibody response to *S. molnari,* with antibodies quantified on western blot; *n* = 5 except multi-strain yeast fraction MsYF (**A**), 28 dpi, where *n* = 3.

Quantification of *S. molnari* by qPCR showed that individuals fed the functional diets had 13 (curcumin) and 32 (MsYF) times lower median values of proliferative blood stages than the control group, at 28 dpi (Figure 4A). Thereby, fish supplemented with curcumin had a consistently low prevalence of *S. molnari* blood stages while the MsYF group had few specimens showing higher infection levels. At 42 dpi, parasite numbers in the blood had decreased in the control diet in comparison to 28 dpi, although the median remained almost the same. Interestingly, at 42 dpi, higher numbers of blood stages were observed in the supplemented diets than in the control, indicating delayed parasite proliferation. Blood parasitemia levels were the highest in the curcumin group, 42 dpi, where the median was three times higher than in the control and 1.5 times higher than in the MsYF group, at 28 dpi. In comparison, blood parasitemia in the MsYF group also increased from 28 to 42 dpi but to similar values as observed in the control diet at that time point. Summarizing the data from both time points, the MsYF group had the overall lowest parasite levels, followed by the curcumin group, while the highest overall parasite quantities were observed in the control group.

Serum antibody responses to *S. molnari* were determined by western blotting (Figure 4B). A 16 kDa parasite protein was recognized by carp IgM in pooled sera of infected carp of each group, 28 and 42 dpi, as previously reported [19]. While the antibody titer, specific to the 16 kDa antigen, was the highest in the control diet at 28 dpi, in accordance with the highest parasite numbers in the blood, a different pattern was observed at 42 dpi. At this time point, the curcumin group had the highest parasitic load but a similar antibody response as the control group. Fish supplemented with MsYF showed a considerably increased specific antibody response despite harboring a similar number of parasites as the control group, 42 dpi.

## 3. Discussion

In the present study, 0.5% curcumin and 0.12% MsYF yeast were used as in-feed prophylactic treatments against infection of common carp with the myxozoan parasite *S. molnari*. Both feed additives altered various immunological parameters in naïve (pre-challenge) and infected fish.

Most importantly, parasite numbers were strongly reduced in both the curcumin and the MsYF group, at 28 dpi in comparison to control. In contrast, at 42 dpi, the highest parasite load in the blood occurred in the curcumin-supplemented group, while the lowest was found in the MsYF group. Hence, the two diets differed considerably with regard to their effects at different stages of the *S. molnari* infection, by acting variably on parasites or the immune response profile.

Curcumin is a potent parasiticidal agent [20], while anti-inflammatory and hepatoprotective properties were ascribed to it in a number of fish and mammalian studies [21,22,23]. In the present study, anti-inflammatory effects in curcumin-fed fish were, characterized by reduced phagocytosis, reactive oxygen species (ROS) and NO production in blood and HK leukocytes, with an increased effect at late-stage infection (42 dpi). At this time point, anti-inflammatory action may have a negative effect on the parasite clearance. This idea is supported by the observation that infection with *S. molnari* induces a massive increase in the expression of the anti-inflammatory il-10 during late stage infection, which may result in a reduced immunocompetence of the infected host, allowing the parasite to persist despite partially acquired immunity [19]. High parasite numbers at 42 dpi in that group also coincided with low NO levels in the HK and it has previously been suggested that NO production plays a beneficial role in the protection against parasitic infections [24]. Thus, in our infection model, curcumin appears to play a dual role; while its parasiticidal effect delays the onset of parasitemia, its anti-inflammatory capacities may reduce the ability of the immune system to control the infection later on.

The MsYF treatment showed strong immunomodulatory properties in naïve carp prior to infection with *S. molnari*, though not characterized by an increase in respiratory burst activity or NO secretion, as suggested by other studies using yeast β-glucans [25]. Despite the fact that most research is focused on the immunostimulatory properties of β-1,3/1,6-glucans of yeast, it is important to note that other polysaccharides are contained in yeast cell wall and that yeast species, strains and production process yield distinct cell composition, architecture and properties [26]. Studies demonstrating enhanced ROS production and NO secretion in response to yeast are mostly limited to in vitro experimental evidence [27,28,29], while we provide proof from an in vivo model. A study in which Atlantic salmon was fed 1% β-glucan failed to show the same effects as in vitro stimulation of cells from control fish [28]. A possible reason for the differences between in vitro and in vivo studies for yeast is the non-digestible nature of β-glucans, although it is suggested that teleosts are capable of β-glucan uptake [25].

In our study, MsYF increased ROS production in the HK only at 42 dpi with *S. molnari* and, importantly, MsYF strongly enhanced the specific antibody response at 42 dpi with sera showing the highest antibody titers measured in this study. As well as an initial reduction in blood parasitemia at 28 dpi, this points towards a significant impact of MsYF also at a later stage of the infection. This could be explained by the combination of distinct yeast strains in MsYF, resulting in the activation of a broad set of immune receptors belonging to microbe-associated molecular patterns (MAMP), including Dectin-1, mannose receptor and Toll-like receptors [30]. Rawling et al. [31] showed that both single-strain yeast fractions and MsYF (composed of two strains of *Saccharomyces cerevisiae* and one strain of *Cyberlindnera jadinii*) improve the intestinal health status and growth performance of European seabass (*Dicentrarchus labrax*), but only MsYF stimulates the innate immune response in a broad specific fashion. Enhanced antibody responses to different pathogen challenges induced by β-glucans has also been observed in other species such as Atlantic salmon (*Salmo salar*) or Channel catfish (*Ictalurus punctatus*) [32,33], supporting the idea that yeast immunomodulatory properties affect not only the innate but also the acquired immune response [30].

Although both experimental diets reduced the initial proliferation of *S. molnari*, the infection was not cleared, and similar or higher numbers of *S. molnari* were detected in the blood in the experimental diets in comparison to the control, at 42 dpi. This could be due to the delayed course of infection or due to parasite evasion strategies such as skewing the host response to an anti-inflammatory phenotype [19]. Despite having different immunomodulatory properties [34], it is likely that myxozoans adopted similar mechanisms to avoid or modulate host immune responses. A number of reports exist showing that specific antibodies are produced against different species, implicating that myxozoans are successfully recognized by the immune system of their host [1,19,35,36,37,38]. Similar as observed in natural infections, feed-additives did not allow carp to eliminate *S. molnari* completely but the multi-strain yeast product in particular had a positive effect on the infection by reducing overall parasite numbers and considerably increasing specific antibody levels. More research with different concentrations and combinations of feed additives would be needed to determine optimum conditions for the use of nutraceuticals against myxozoans in aquaculture, but the present study shows that yeast-based products can strongly influence the course of myxozoan diseases, as they appear to impact beneficially at early and later stage infections, by enhancing both innate and adaptive immune mechanisms.

## 4. Materials and Methods

### 4.1. Isolation of S. Molnari Blood Stages

Proliferative blood stages of *S. molnari* were obtained from the blood of donor fish at the peak of infection using adapted diethylaminoethyl-cellulose isolation as described previously [39]. Briefly, blood from infected carp was collected, using a heparinized syringe, and added to an equal volume of RPMI 1640 medium (Life Technologies, Prague, Czech Republic), containing 50 U/mL of Heparin. Following density centrifugation at 800 g for 20 min, at 4 °C using Ficoll, the parasite and blood leukocytes were collected from the interphase and parasites were subsequently separated from fish leukocytes on cellulose column. The enriched parasite (purity ˃ 95%) was pelleted and adjusted to 1 × 10^6^ parasites mL^–1^.

### 4.2. Experimental Design

Specific pathogen-free (SPF) common carp (*Cyprinus caprio*) (*n* = 120; body weight = 105 g; age < 2 years) were kept in a 30 m^3^ indoor recirculation aquaculture system, divided into three groups held in 600 L tanks (40 fish/tank) under 12L:12D photoperiod, at a constant 21 °C water temperature. Due to the pilot character of the study, no replicate tanks were included. Water quality (oxygen, pH, ammonia, nitrite, nitrates) was monitored on a daily basis using probes and titration tests. During the trial, fish were fed with three diets—a carp base diet with no additives (Europe15; Skretting, Stavanger, Norway), the same base diet enriched with 0.5% curcumin (Sigma-Aldrich, Prague, Czech Republic) and enriched with 0.12% MsYF (Lallemand, Blagnac, France), at a daily rate of 2% of biomass. MsYF is an inactivated yeast-based product consisting of two strains of *Saccharomyces cerevisiae* and one strain of *Cyberlindnera jadinii*, whose bio-morphological characteristics are provided by Rawling et al. [31]. All diets were produced by heat extrusion (Skretting ARC, Stavanger, Norway) and stored at 4 °C thereafter and during the feeding study. Two months after the start of the trial, 10 fish per group were individually tagged with glass transponders (AEG) and three groups were injected intraperitoneally with 150,000 *S. molnari* blood stages.

### 4.3. Sampling

To evaluate the effects of the diets on immune system, fish were sampled after two months of feeding, and thereafter at the peak of blood and liver parasitemia, at 28 days post infection (dpi) and 42 dpi, as described previously [19]. Unless stated otherwise in the figure legends, at each time point five fish per group were sampled for all immunological and parasitological investigations described below. One ml of blood was collected from anesthetized fish (100 ppm of clove oil) using a sterile, heparinized syringe and diluted in 1 mL of RPMI 1640 medium, containing 50 U/mL of heparin. Another ml of whole blood was taken using a distinct sterile, heparinized syringe to collect plasma after centrifugation (10,000 g, 5 min), while 4 µL of full blood were stored in RNA protect (Quiagen, Hilden, Germany) for quantification of the parasite stages by qPCR. For subsequent cells assays, a piece of head kidney (HK) was teased through a sterile 100 µm nylon mesh, using RPMI 1640 medium. To prevent DNA cross contamination, 10% hydrogen peroxide was used routinely to clean scissors and tweezers during sampling. 

Fish body weight was assessed at the start of the trial and at 42 dpi when the 106-day trial was complemented. All animal procedures were performed in accordance with Czech legislation (section 29 of Act No. 246/1992 Coll., on Protection of animals against cruelty, as amended by Act No. 77/2004 Coll.). Animal handling hence complied with the relevant European guidelines on animal welfare (Directive 2010/63/EU on the protection of animals used for scientific purposes) and the recommendations of the Federation of Laboratory Animal Science Associations.

### 4.4. Isolation of Leukocytes from Blood and HK

For the isolation of leukocytes from blood, 1 mL of heparinized blood diluted in RPMI 1640 medium was loaded onto a 57% Percoll gradient, while HK leukocytes were isolated using a discontinuous 34%/57% Percoll gradient to clear the remaining cell debris. After centrifugation at 400 g for 30 min at 4 °C, the cells at the interphase were collected, washed in RPMI 1640 medium and centrifuged at 400 g for 6 min. For subsequent cell assays, blood and HK leukocytes were counted in a Bürker chamber.

### 4.5. Respiratory Burst

Respiratory burst activity was determined by the reduction of nitro-blue tetrazolium (NBT) by intracellular 02- (NBT assay) in HK leukocytes. Briefly, cells were seeded onto a 96 well plate with a density of 1 × 10^6^ cells per well and subsequently stimulated with phorbol 12-myristate 13-acetate (PMA) Following incubation with 5% CO_2_ at 25 °C for 2 h, cells were washed with 1640 RPMI medium (without phenol red) and fixed with 100% methanol for 5 min. Crystals were resuspended with 120 μL 2M KOH and 140 μL DMSO, and absorbance was measured at 690 nm with a reference filter of 414 nm, using a spectrophotometer.

### 4.6. Phagocytosis

Phagocytic activity of host leukocytes was evaluated in both blood and head kidney leukocytes, using a protocol previously described [40]. Blood and HK leukocytes were seeded onto a 96 well plate at a concentration of 1 × 10^6^ cells per well. Fluorescent beads (aqueous suspension, 1.0 μm mean particle size, Sigma-Aldrich, Prague, Czech Republic; 5 μL/mL) were mixed with RPMI medium (Life Technologies, Prague, Czech Republic) containing 5% heat-treated carp serum (63 °C, 10 min), 5% fetal calf serum (PAA Laboratories GmbH, Pasching, Austria) and 1% penicillin/streptomycin (Sigma-Aldrich, Prague, Czech Republic) and incubated at 5% CO2 at 25 °C, for 4 h. After incubation, cells were washed twice with RPMI medium and the proportion of phagocytic cells was measured by flow cytometry.

### 4.7. Nitric Oxide (NO) Quantification

NO production by carp HK leukocytes was evaluated using a Nitric Oxide detection kit. Briefly, freshly isolated leukocytes were added to a 96 well plate (1 × 10^6^ cells per well) and incubated in RPMI medium (including 5% naïve carp serum, 5% FCS and 1% streptomycin/penicillin), at 25 °C with 5% CO_2_, for 24 h. Following incubation, the plate was spun at 400 g for 3 min and the supernatant was transferred to a new plate and the NO concentration was measured according to manufacturer instructions (Nitric Oxide (total) detection kit; Enzo Life Sciences, Lausen, Switzerland).

### 4.8. B cell Numbers

Blood leukocytes were seeded onto a 96 well v-bottom plate at a density of 1 × 10^6^ cells per well. Cells were immunolabelled with a primary monoclonal mouse anti-carp IgM antibody (Aquatic Diagnostics, Stirling, UK; 400 ng per sample) and a secondary goat anti-mouse, Alexa Fluor 488-labelled antibody (1:1000; Life Technologies, Prague, Czech Republic). The proportion of IgM+ B cells in the leukocyte population was measured by flow cytometry.

### 4.9. S. molnari Quantification

Blood in RNAprotect was thawed and centrifuged 10 min at 6000× *g*. The supernatant was carefully removed and 50 µL of sterile PBS added to the cells before admixing 350 µL of TNES urea buffer [41]. Thereafter, 100 µg/mL proteinase K was added and blood samples were left to digest overnight, at 55 °C. DNA was extracted using a modified phenol-chloroform protocol [42]. Parasite quantification was performed using TaqMan qPCR. Parasites were quantified based on the number of genomic SSU rDNA copies relative to carp β-actin as a reference gene [19].

### 4.10. Detection of S. molnari-Specific Antibodies

Specific serum antibody responses to S. molnari were determined by western blot as described previously [19]. Parasite isolates in PBS (1600 BS/μL) were diluted 1:1 with Laemmli Sample buffer (Bio-Rad, Prague, Czech Republic) with 5% 2-mercaptoethanol (Sigma-Aldrich, Prague, Czech Republic). The sample was boiled for 5 min and nine replicas (8000 BS) were run on sodium dodecyl sulfate polyacrylamide gels and electro-phoretically transferred onto a Trans-Blot^®^ Turbo™ Mini-size polyvinylidene difluoride membranes (Bio-Rad, Prague, Czech Republic). The membranes were blocked in 5% nonfat dry milk (Bio-Rad, Prague, Czech Republic) with 0.1% Tween-20 in PBS for 1 h. Nine different lanes were cut into strips and each was incubated for 1 h with pooled sera from five fish of each diet (1:100 dilution), obtained on both sampling dates (28 dpi and 42 dpi). The membrane was then incubated for 1 h with a monoclonal mouse anti-carp IgM antibody (1:5000; 2000 ng) and a HRP (horseradish peroxidase)-labelled goat anti-mouse antibody (1:5000), for 30 min. Blots were developed with an enhanced chemiluminescence substrate (Bio-Rad, Prague, Czech Republic) and recorded with a ChemiDoc imaging station (Bio-Rad). Intensity of the bands was quantified using the Image Lab software (Bio-Rad, Prague, Czech Republic).

### 4.11. Statistics

Results from all assays were tested for statistically significant differences between control and experimental diets using the Kruskal-Wallis Test where *p*-values < 0.05 were considered significant.

## 5. Conclusions

Due to commercial expansion and climate change, parasitic diseases in aquaculture are emerging, and will emerge further, in the near future. Although vaccine development against fish pathogens is rapidly progressing, these specific treatments exist only against bacterial and viral pathogens and there are currently no available vaccines against fish parasites [38]. Natural compounds can offer complementary advantages over other preventive health practices since their administration does not require major interventions, neither do they lead to disease resistance. In our study, both curcumin and MsYF postponed parasite proliferation in the blood and enhanced specific antibody responses, with MsYF demonstrating a highly beneficial modulation of the entire course of infection. The present study suggests specific yeast products are powerful tools for improving host immune responses towards myxozoans and providing important health benefits during their infection.
